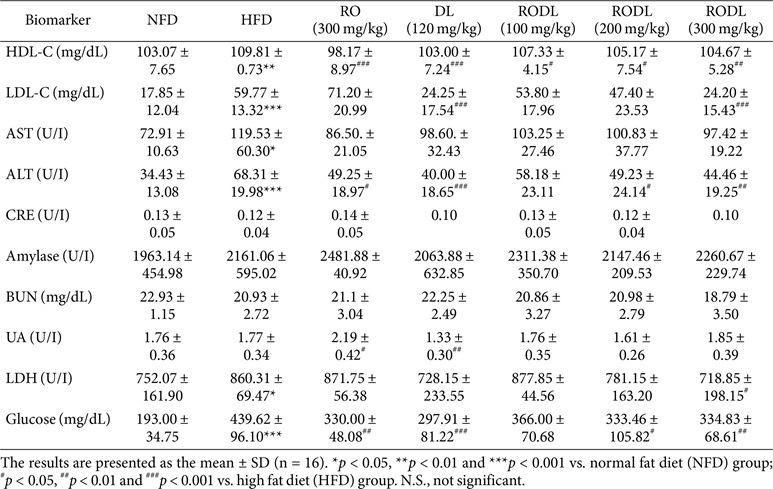# Dual Mechanistic Anti-Obesity Effects of Red Okra and *Diospyros lotus* Mixture via Fat Binding and AMPK-Mediated Lipid Metabolism

**DOI:** 10.4014/jmb.2506.06030

**Published:** 2025-09-24

**Authors:** Yeo-Jin Choi, Wan Seok Kang, Eun Kim, Seon ah Son, Ki Hoon Lee, Youngbae Kim, Jin Seok Kim, Sunoh Kim

**Affiliations:** Central R&D Center, B&Tech Co., Ltd., Naju 58205, Republic of Korea

**Keywords:** Red okra, *Diospyros lotus*, obesity, lipid metabolism, AMPK, fat binding capacity

## Abstract

Obesity is a major global health issue associated with metabolic dysfunctions including dyslipidemia, insulin resistance, and hepatic steatosis. This study investigated the dual anti-obesity mechanisms of a combined extract of red okra (*Abelmoschus esculentus* L. Moench, RO) and *Diospyros lotus* L. leaves (DL) in a high-fat diet (HFD)-induced obese mouse model. Fat-binding capacity (FBC) analysis revealed that RO exhibited significantly higher FBC than DL, suggesting a greater potential to inhibit intestinal fat absorption. *In vitro*, RO more effectively suppressed lipid accumulation in 3T3-L1 adipocytes, whereas DL enhanced lipolytic activity by stimulating glycerol release from differentiated adipocytes. To optimize the complementary functions of both extracts, various mixing ratios were evaluated, and the 4:1 ratio (RODL^TM^) was identified as optimal. This combination resulted in approximately 25% reduction in lipid accumulation, a significant increase in glycerol release, and a 39% elevation in fecal fat excretion. In HFD-fed mice, oral administration of the RODL mixture led to up to 14% suppression in body weight gain, improved serum triglyceride and cholesterol levels, and alleviated hepatic steatosis. Mechanistically, the extract combination activated AMPKα signaling, leading to the upregulation of lipolysis-related proteins (ATGL, pHSL), thermogenesis-related markers (UCP1, PGC1α), and fatty acid oxidation regulators (PPARα, CPT1, ACOX1). These findings indicate that the RO and DL extract combination exerts anti-obesity effects through both inhibition of fat absorption and modulation of lipid metabolic pathways. This dual mechanism supports its potential as a safe and effective natural therapeutic for obesity prevention and management.

## Introduction

Obesity is a multifactorial metabolic disorder characterized by excessive fat accumulation and is associated with an increased risk of insulin resistance, dyslipidemia, type 2 diabetes, and cardiovascular disease. Given the complex pathophysiology of obesity, therapeutic strategies that target a single mechanism often yield limited efficacy. Consequently, there is growing interest in multi-targeted approaches that address multiple aspects of lipid metabolism and energy homeostasis [1]. Sustainable obesity management has led to the development of diverse interventions, including structured weight management programs, exercise regimens, dietary modifications, functional foods, and pharmacological treatments. Among these, three key mechanisms have emerged as essential targets in obesity control: (i) inhibition of dietary fat absorption via physical binding of lipids in the gastrointestinal tract, thereby reducing caloric intake and enhancing fecal fat excretion; (ii) suppression of preadipocyte differentiation into mature adipocytes, which limits adipose tissue expansion; and (iii) stimulation of lipolysis, the hydrolysis of triglycerides stored in adipocytes, which facilitates fatty acid mobilization and energy utilization [2, 3]. These mechanisms act synergistically to reduce lipid accumulation and promote energy expenditure, making them attractive targets for functional food ingredients and botanical therapeutics [4].

Accordingly, combination formulations that concurrently inhibit lipid absorption, suppress adipogenesis, and activate lipolysis represent a promising strategy for comprehensive obesity management. In this context, natural products with multifunctional bioactive properties are under active investigation as safe and effective alternatives to synthetic anti-obesity drugs. Their mechanisms of action include modulation of host metabolism, promotion of thermogenesis, appetite regulation, inhibition of digestive enzymes such as pancreatic lipase and amylase, improvement of insulin sensitivity, suppression of adipogenesis, and induction of adipocyte apoptosis. These diverse bioactivities make natural compounds strong candidates for development into effective botanical therapies for obesity. In the present study, we aimed to develop a multifunctional herbal extract combination capable of inhibiting fat accumulation, promoting lipolysis, and enhancing fat excretion. Through this, we sought to improve the overall efficacy of obesity management and provide a safer and more practical therapeutic strategy.

Okra (*Abelmoschus esculentus* L. Moench), a flowering plant in the Malvaceae family, is traditionally consumed as a vegetable across Africa and South Asia and is now gaining global popularity [5, 6]. It has been used in traditional medicine to treat constipation, leukemia, diabetes, and jaundice [7]. Okra pods and seeds are nutrient-dense, containing essential fatty acids, proteins, amino acids, vitamins, andminerals [8]. The seeds are particularly rich in oil (up to 40%), especially linoleic acid, a polyunsaturated fatty acid. They also contain high-quality proteins with favorable amino acid profiles, including lysine and tryptophan. Okra pods are excellent sources of dietary fiber, β-carotene, B vitamins, and keyminerals such as calcium, magnesium, and iron. Moreover, the mucilage from okra pods, rich in polysaccharides, contributes to cholesterol-lowering effects. Among various cultivars, red okra is notable for its vivid pigmentation, primarily due to anthocyanins and other polyphenolic compounds [9, 10]. These phytochemicals not only confer antioxidant properties but may also offer superior health benefits compared to green okra. Additionally, the pectic substances in okra may inhibit lipid digestion and absorption by binding to calcium ions and bile acids and by obstructing lipase access to micellar lipid droplets, essential for lipid assimilation [11].

*Diospyros lotus* L., native to tropical and subtropical regions, is a medicinal plant widely cultivated for its edible fruits [12]. Traditionally, various parts of the plant, fruits, leaves, and roots, have been used to treat constipation, serve as a sedative, and exert antidiabetic, antitumor, laxative, febrifuge, and nutritive effects [13-15]. In particular, leaf extracts of *D. lotus* are known to contain diverse phytochemicals, including gallic acid, kaempferol, myricetin, and their derivatives, as well as multiple fatty acids, sugars, and organic acids [16, 17].

In this study, we investigated the individual effects of red okra pod extract (RO) and *D. lotus* leaf extract (DL) on lipid metabolism and identified an optimal combination ratio that maximized their complementary anti-obesity effects. Based on this optimized formulation, we developed a synergistic extract and elucidated its mechanism of action *in vivo* using a high-fat diet (HFD)-induced obese mouse model.

## Materials and Methods

### Reagents

The 3T3-L1 preadipocyte cell line (CL-173) was purchased from the American Type Culture Collection (ATCC, USA). Cells were maintained in Dulbecco’s Modified Eagle’s Medium (DMEM; containing D-glucose, L-glutamine, sodium pyruvate, and sodium bicarbonate), supplemented with 10% bovine calf serum (BCS) and 1%penicillin-streptomycin (P/S), all obtained from Invitrogen, Inc., (USA). 3-Isobutyl-1-methylxanthine (IBMX), dexamethasone (DEX), insulin, and Oil Red O (ORO) were purchased from Sigma-Aldrich (USA).

### Extraction of Red Okra and *Diospyros lotus* L. Leaves

Red okra seeds (Candle Fire hybrid F1, non-GMO) were obtained from KNOWN-YOU SEED Co., Ltd.(Taiwan). Red okra and *D. lotus* were cultivated in 2022 in Gangjin-gun, Jeollanam-do, Republic of Korea, and authenticated by Dr. Kim at the R&D Center of B&Tech Co., Ltd. (Republic of Korea). Harvested red okra pods and *D. lotus* leaves were washed, cut into 1 cm segments, and dried using hot air at 55°C for 30 h (DY-280H, Daeyoung E&B, Republic of Korea). Dried plant materials were extracted with distilled water at 100°C for 6 h. The resulting extracts were filtered, concentrated under reduced pressure using a rotary evaporator, and vacuum-dried. The final powder extracts (red okra, RO; *D. lotus* leaves, DL) were reconstituted in 0.9% saline for *in vivo* experiments and in distilled water for *in vitro* experiments.

### HPLC Analysis of Quercetin-3-O-Gentiobioside and Myricetin

Quantitative analysis of quercetin-3-O-gentiobioside and myricetin was performed using a high-performance liquid chromatography (HPLC) system (Agilent 1260 Infinity, Agilent Technologies, USA) equipped with a diode array detector (DAD). Chromatographic separation was achieved using an Eclipse XDB-C18 column (4.6 × 150 mm, 5 μm particle size) maintained at 40°C. The mobile phase consisted of solvent A (0.5% acetic acid in distilled water, v/v) and solvent B (100% acetonitrile), delivered at a flow rate of 1.0 ml/min. The injection volume for each sample was 10 μl.

For quercetin-3-O-gentiobioside, gradient elution was performed as follows: 5% solvent B was held for the initial 5 min, increased linearly to 20% B over the next 45 min (5–50 min), returned to 5% B within 1 min (50–51 min), and maintained for re-equilibration at 5% B for 4 min (51–55 min). Detection was carried out at a wavelength of 354 nm.

For myricetin, a different gradient program was applied: solvent B was increased from 17% to 23% over 15 min (0–15 min), then further increased to 60% B from 15 to 25 min. The gradient was returned to 17% B over 1 min (25–26 min), followed by re-equilibration at 17% B for 4 min (26–30 min). The detection wavelength for myricetin was set at 370 nm.

Compounds were identified by comparing retention times and UV absorption spectra with those of authentic standards (purity ≥ 98%). Quantification was performed using calibration curves constructed from serial dilutions of standard solutions. The calibration curve for quercetin-3-O-gentiobioside (5–100 ppm) showed excellent linearity with R^2^ = 1.0000. For myricetin, a linear range of 10–80 ppm was used with R^2^ = 0.9999. All analyses were performed in triplicate for each of the five independently prepared extract batches, and the final concentrations of quercetin-3-O-gentiobioside and myricetin in the samples were expressed as milligrams per gram of dried extract (mg/g).

### Cell Culture

3T3-L1 preadipocytes were cultured in high-glucose DMEM supplemented with 10% BCS and 100 U/ml P/S at 37°C in a humidified atmosphere containing 5% CO_2_. Upon reaching confluence, the cells were induced to differentiate by treating with complete medium containing 0.5 mM IBMX, 1 μM DEX, and 5 μg/ml insulin for 2 days. Subsequently, the cells were cultured for an additional 2 days in complete medium containing 5 μg/ml insulin. Thereafter, the differentiation medium was replaced with standard complete medium, and cells were maintained with medium changes every 2 days until full differentiation was achieved. Full differentiation was defined as the point at which ≥80% of cells displayed cytoplasmic lipid droplets detectable by ORO staining, accompanied by characteristic morphological rounding of the cells.

### Lipid Accumulation

To evaluate the effects of RO, DL, and their combinations on lipid accumulation, 3T3-L1 preadipocytes were cultured in differentiation medium containing RO, DL, or RODL mixtures at concentrations ranging from 0 to 100 μg/ml, or at various mixing ratios (1:1, 2:1, 3:1, 4:1, 1:2, 1:3, and 1:4) for 7 days. Mature adipocytes were also treated with RO, DL, or RODL combinations at equivalent concentrations and ratios for 3 days in complete medium. Total intracellular lipid content was assessed using ORO staining. Cells were fixed in 4% formaldehyde for 20 min, rinsed with 60% isopropanol, and air-dried completely. The cells were then stained with ORO solution for 30 min at room temperature and imaged using an inverted microscope (Nikon Instruments, USA). To quantify intracellular lipid content, the dye retained in the cells was eluted with 100% isopropanol, and the optical density (OD) of the extracted solution was measured at 490 nm using a microplate reader.

### Fat Binding Capacity

The fat binding capacity (FBC) was determined according to the method described by Rasweefali *et al*. (2021), withminor modifications [18]. Briefly, 1 g of RO, DL, or RODL combination was placed into a pre-weighed centrifuge tube. Subsequently, 10 ml of either soybean oil or lard was added to each tube. The mixture was gently stirred every 10 min over a 30 min period to ensure thorough interaction between the extract and lipid phase. After incubation, the samples were centrifuged at 3,000 ×*g* for 15 min at room temperature. The supernatant was carefully removed, and the residual pellet was weighed. FBC was calculated as the increase in weight compared to the initial powder weight, reflecting the amount of oil retained by the sample. All measurements were performed in triplicate, and FBC values were expressed as percentages (%).

### Measurement of Free Glycerol Release

Fully differentiated 3T3-L1 adipocytes were gently washed once with phosphate-buffered saline (PBS) and incubated for 24 h in phenol red- and serum-free DMEM containing the RODL combination at concentrations ranging from 0 to 100 μg/ml. Following incubation, the amount of glycerol released into the culture medium was measured using an enzymatic colorimetric assay kit (Cayman Chemical Co., USA) according to the manufacturer’s instructions. Glycerol concentration was expressed as micrograms per milliliter of culture medium (μg/ml).

### Animal and Experimental Design

Male Sprague Dawley (SD) rats (6 weeks old) and specific pathogen-free (SPF) grade male C57BL/6 mice (4 weeks old) were purchased from Samtako Bio Korea (Republic of Korea). All animals were acclimated for one week under standard housing conditions. Rats were randomly assigned to groups of 10 animals each and administered lard and extracts by oral gavage. The experimental groups included: (i) normal control (CTL; distilled water), (ii) lard-only control (Lard; lard + distilled water), and (iii) treatment groups receiving lard plus 100, 150, or 300 mg/kg of either RO, DL, or the RODL combination (4:1 ratio). Blood samples were collected 4 h after oral administration and centrifuged at 1,000 ×*g* for 15 min at 4°C to obtain serum. Fresh fecal samples were collected 24 h after administration and stored at −80°C until analysis.

For the mouse experiment, animals were fed either a normal-fat diet (NFD; 10% kcal from fat) or a HFD (60%kcal from fat) obtained from Research Diets (D12450B and D12492, New Brunswick, USA). After 10 days of HFD induction, mice were randomly assigned to groups of 16 animals and orally administered RODL (100, 200, or 300 mg/kg), RO alone (300 mg/kg), or DL alone (120 mg/kg) daily for 8 weeks. All treatments were administered during continued HFD feeding. Body weight was measured every 2–3 days. At the end of the experimental period, mice were sacrificed for collection of blood, organ tissues, and fecal samples for further analysis.

All animal procedures were conducted in accordance with the International Guidelines for the Care and Use of Laboratory Animals and approved by the Institutional Animal Care and Use Committee (IACUC) of Bioresources and Technology Co., Ltd. (B&Tech, Republic of Korea), in compliance with the Animal Protection Act of the Republic of Korea (Approval numbers: BT-001-2021 and BT-003-2021).

### Analysis of Plasma Lipid Profiles

At the end of the 67-day experimental period, mice were sacrificed under isoflurane anesthesia. Blood was collected and centrifuged at 1,000 ×*g* for 15 min at 4°C, and the resulting serum was stored at −80°C until further analysis. After blood collection, white adipose tissues (WAT), brown adipose tissue (BAT), and liver were immediately dissected and weighed. Plasma levels of high-density lipoprotein cholesterol (HDL-C, mg/dL), low-density lipoprotein cholesterol (LDL-C, mg/dL), blood urea nitrogen (BUN, mg/dL), and glucose (mg/dL) were measured using the Cholestech LDX System (Alere Inc., USA). Serum concentrations of triglycerides (TG, mg/dL), total cholesterol (TC, mg/dL), aspartate aminotransferase (AST, U/L), and alanine aminotransferase (ALT, U/L) were quantified using commercial assay kits (Asan Pharm, Republic of Korea). Additionally, creatinine (CRE, U/L), amylase (U/L), uric acid (UA, U/L), and lactate dehydrogenase (LDH, U/L) levels were measured using an automated biochemical analyzer (DRI-CHEM NX500i, Fujifilm, Japan) with corresponding diagnostic kits.

### Histological Analysis

Liver tissues were excised and cut into small pieces, then fixed in 4% formalin solution for 24 h at room temperature. Fixed samples were embedded in paraffin, sectioned into 5 μm-thick slices, and stained with Mayer’s hematoxylin and eosin (H&E) for histological evaluation. Stained sections were visualized under an optical microscope (Olympus BX51, Japan), and image analysis was performed using MetaMorph microscopy automation and image analysis software (Molecular Devices, USA).

### Protein Extraction and Western Blot Analysis

Epididymal white adipose tissue (eWAT), inguinal white adipose tissue (iWAT), and brown adipose tissue (BAT) were homogenized and lysed in RIPA buffer (Thermo Fisher Scientific, USA) supplemented with a complete protease inhibitor cocktail (cOmplete Tablets, Switzerland). Total protein concentrations were determined using the Bradford assay with bovine serum albumin (BSA) as the standard.

Equal amounts of protein were resolved by 8–12% SDS–polyacrylamide gel electrophoresis (SDS–PAGE) and transferred onto polyvinylidene difluoride (PVDF) membranes (EMD Millipore, USA). Membranes were incubated overnight at 4°C with primary antibodies, followed by incubation with horseradish peroxidase (HRP)-conjugated secondary antibodies for 1h at room temperature. Detection was performed using enhanced chemiluminescence (ECL) reagents (Millipore Corporation), and protein bands were visualized with a ChemiDoc XRS+ imaging system (Bio-Rad, USA). Band intensities were normalized to β-actin and quantified using ImageJ software (NIH, USA). The primary antibodies used in this study were obtained from multiple suppliers. Antibodies purchased from Cell Signaling Technology (USA) included anti-phospho-AMP-activated protein kinase α (pAMPKα, 1:1000, #2531), anti-AMP-activated protein kinase α (AMPKα, 1:1000, #2532), anti-phospho-hormone-sensitive lipase (pHSL, 1:500, #45804), anti-hormone-sensitive lipase (HSL, 1:1000, #4107), anti-perilipin 1 (PLIN1, 1:1000, #3467), anti-uncoupling protein 1 (UCP1, 1:1000, #14670), anti-phospho-acetyl-CoA carboxylase 1 (pACC1, 1:1000, #3661), anti-acetyl-CoA carboxylase 1 (ACC1, 1:1000, #3662), and anti-fatty acid synthase (FASN, 1:1000, #3189). Antibodies from Santa Cruz Biotechnology (USA) included anti-adipose triglyceride lipase (ATGL, 1:1000, #365278), anti-peroxisome proliferator-activated receptor γ coactivator 1-α (PGC1α, 1:500, #518025), anti-carnitine palmitoyltransferase 1 (CPT1, 1:500, #393070), and anti-β-actin (1:2000, #47778). Additionally, anti-phospho-perilipin 1 (pPLIN11:1000, #4856) was purchased from VALA Sciences (USA), and anti-acyl-CoA oxidase 1 (ACOX1, 1:1000, #184032) was obtained from Abcam (USA). Secondary antibodies (1:5000 dilution) were purchased from Cell Signaling Technology.

### Statistical Analysis

Statistical analyses were performed using Student’s *t*-test or one-way analysis of variance (ANOVA) followed by Tukey’s post hoc test for multiple comparisons. Normality and homogeneity of variance were evaluated using the Shapiro–Wilk and Levene’s tests, respectively. All data are expressed as mean ± standard deviation (SD). Statistical significance was set at *p* < 0.05. All analyses were conducted using GraphPad Prism version 5 (GraphPad Software, USA).

## Results

### Effects of RO and DL on Fat Binding Capacity

**Fig. 1:**
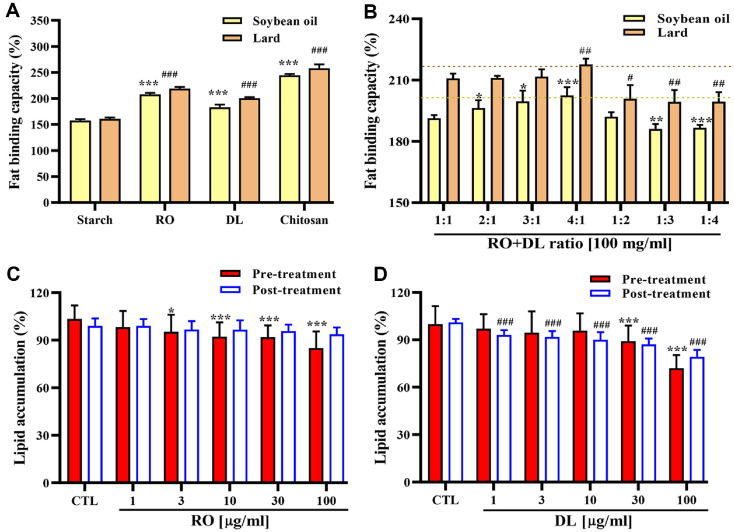
Effects of RO and DL on fat binding capacity and lipid accumulation. (**A**) Fat binding capacity (FBC) of 100 mg/ml of red okra (RO) and *D. lotus* (DL) was independently measured using soybean oil or lard as lipid sources. Starch was used as a negative control, while chitosan was included as a positive control for physical fat binding. (**B**) FBC of RO and DL combinations at various mixing ratios (*w/w*) was assessed using soybean oil and lard. (**C, D**) Preadipocytes and matured 3T3- L1 adipocytes were incubated with various concentrations of RO (**C**) and DL (**D**), and intracellular lipid accumulation was quantified by measuring optical density at 490 nm following Oil Red O (ORO) staining. All data are presented as mean ± SD from at least three independent experiments, each performed in triplicate. *, ^#^*p* < 0.05, **, ^##^*p* < 0.01 and ***, ^###^*p* < 0.001 versus starch, 1:1 or control (CRL).

The FBC of RO and DL, as well as their combinations, was evaluated using soybean oil and lard as model lipids. Starch was used as a negative control, while chitosan was included as a positive control for physical fat binding. Both RO and DL demonstrated significantly higher FBC compared to starch, with RO exhibiting superior FBC relative to DL for both lipid types (Fig. 1A). To investigate the optimal extract ratio for maximizing FBC, various volume ratios of RO and DL were tested. FBC increased as the proportion of RO increased, while higher DL content resulted in a gradual decrease in FBC. Among all combinations, the 4:1 (RODL) ratio showed the highest FBC in both soybean oil and lard (Fig. 1B). These results indicate that RO contributes predominantly to intestinal lipid sequestration through physical binding mechanisms.

### Effects of RO and DL on Lipid Accumulation

**Fig. 2:**
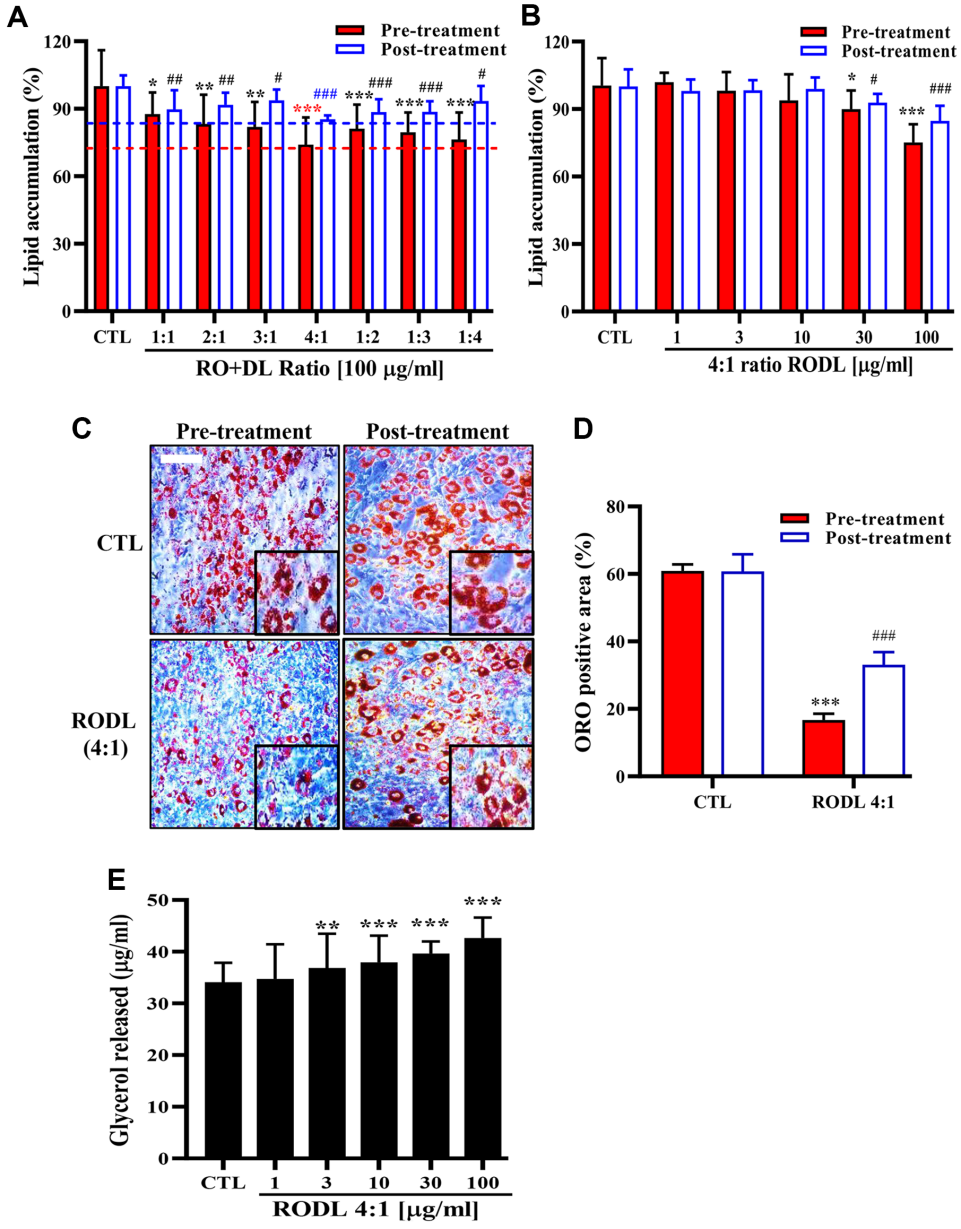
Inhibitory effects of RO and DL mixture on lipid accumulation and stimulatory effects on glycerol release in 3T3-L1 adipocytes. (**A**) Preadipocytes and mature 3T3-L1 adipocytes were treated with varying ratios of red okra (RO) and *D. lotus* (DL) to assess the optimal combination for lipid reduction. (**B**) Preadipocytes and mature 3T3-L1 adipocytes were treated with increasing concentrations of the RODL mixture at a fixed 4:1 ratio, and intracellular lipid content was quantified by Oil Red O (ORO) staining. (**C**) Representative images of ORO-stained preadipocytes and mature adipocytes were obtained at 200× magnification (scale bars represent 100 μm) and lipid droplet area (μm^2^) was quantified using ImageJ software (**D**). (**E**) To evaluate lipolytic activity, mature 3T3-L1 adipocytes were incubated with RODL (4:1) for 24 h, and free glycerol release into the culture medium was measured as an indicator of triglyceride (TG) breakdown. Data are presented as mean ± SD from at least three independent experiments. Statistical significance is indicated as follows: *p < 0.05, **p < 0.01, ***p < 0.001 vs. untreated control (CTL); ^#^*p* < 0.05, ^##^*p* < 0.01, ^###^*p* < 0.001 vs. 1:1 ratio group.

The effects of RO and DL on lipid accumulation were investigated in 3T3-L1 adipocytes. Prior to treatment, MTT (3-(4,5-dimethylthiazol-2-yl)-2,5-diphenyltetrazolium bromide) assay confirmed that both extracts exhibited no cytotoxicity up to 200 μg/ml, supporting their safety for use in *in vitro* adipocyte models (Fig. S1). To assess anti-lipogenic activity, RO or DL was added during the differentiation of preadipocytes to adipocytes. ORO staining demonstrated that both extracts significantly inhibited intracellular lipid accumulation in a dose-dependent manner (Fig. 1C and 1D). For evaluation of lipolytic activity, mature adipocytes were treated with the extracts. DL treatment resulted in a concentration-dependent reduction in lipid content, whereas RO had no significant effect under the same conditions. To examine synergistic ratio, various RODL ratios were tested (Fig. 2A). Among them, the 4:1 (RODL) combination exhibited the greatest reduction in lipid accumulation. Treatment with 80 μg/ml RO + 20 μg/ml DL (100 μg/ml, 4:1 RODL) reduced lipid accumulation to 76.32 ± 12.03%, *p* < 0.001), which was significantly lower than RO alone at 100 μg/ml (85.09 ± 10.46%, *p* < 0.001, Fig. 1C). In mature adipocytes, the same combination resulted in a dose-dependent reduction in lipid accumulation, with an approximate 14% decrease observed in differentiating cells at the highest concentration (*p* < 0.001) (Fig. 2B). Microscopic analysis confirmed a marked decrease in both the number and size of ORO-stained lipid droplets in preadipocytes (pre-treatment) and mature adipocytes (post-treatment) treated with the 4:1 RODL combination (Fig. 2C and 2D). These findings suggest that RO primarily inhibits lipid accumulation during adipogenesis, while DL facilitates lipid degradation in mature adipocytes, indicating complementary mechanisms of action. Furthermore, to confirm enhanced lipolysis, free glycerol release was measured after 24 h treatment with the 4:1 RODL combination. Glycerol levels in the culture medium increased in a dose-dependent manner, supporting the role of the combination in enhancing triacylglycerol breakdown (Fig. 2E). Collectively, these results demonstrate that RO is more effective in preventing lipid absorption through direct fat binding, whereas DL exerts its effects through intracellular lipolysis. The 4:1 combination effectively harnesses both mechanisms, resulting in dual anti-obesity action via inhibition of fat absorption and modulation of adipocyte lipid metabolism.

### RO and/or DL Reduce Blood TG and Promote Fecal Fat Excretion in Lard-Fed Rats

We hypothesized that the FBC of each extract would influence intestinal fat absorption, thereby altering circulating TG levels and fecal fat excretion. To test this, rats were orally administered RO, DL, or a RODL (4:1) mixture at doses of 100, 150, or 300 mg/kg, along with 2 ml of lard. Blood TG levels were measured 4 h post-administration, and fecal fat content was quantified after 24 h.

**Fig. 3:**
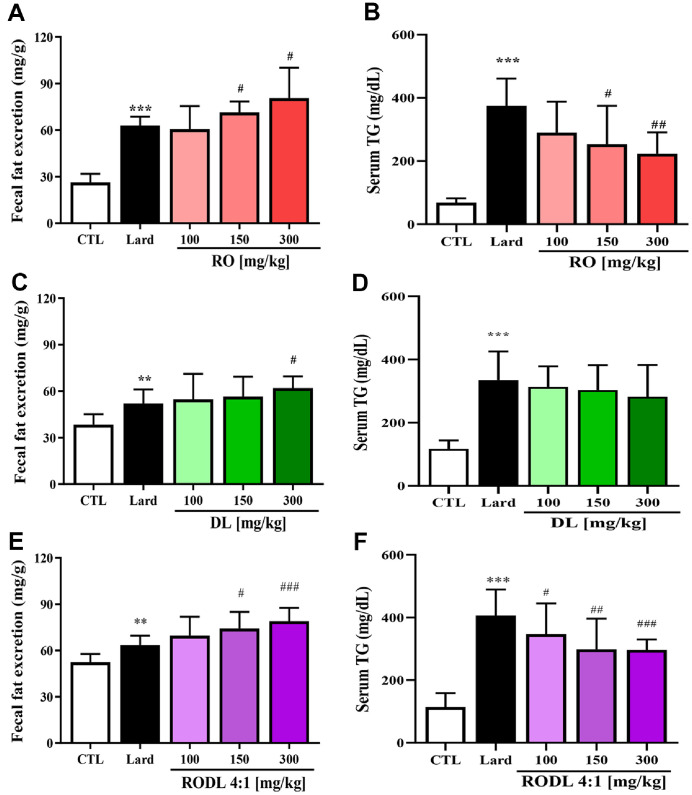
Effects of RO and DL on fecal fat excretion and serum triglyceride levels in lard-fed SD rats. Red okra (RO), *D. lotus* (DL), or their combination (RO:DL = 4:1) was orally administered to SD rats at doses of 100, 150, or 300 mg/kg, along with 2 ml of lard. Blood samples were collected 4 h post-administration to measure serum triglyceride (TG) levels, and feces were collected after 24 h to determine total fecal fat content. (**A**) and (**B**) show the effects of RO alone on fecal fat excretion and serum TG levels, respectively. (**C**) and (**D**) present the corresponding results for DL. (**E**) and (**F**) illustrate the combined effect of RO and DL (4:1) on fecal fat and TG levels. Data are expressed as mean ± SD, *n* = 10 per group. Statistical significance is indicated as *p < 0.05, **p < 0.01, ***p < 0.001 vs. normal diet group (CTL); ^#^*p* < 0.05, ^##^*p* < 0.01, ^###^*p* < 0.001 vs. lard-only control group (Lard).

In RO-treated rats, fecal fat excretion increased in a dose-dependent manner (Fig. 3A). Compared to the lard-only control group (59.5 mg/g, *p* < 0.001), fecal fat levels increased by 13% (*p* < 0.05) at 150 mg/kg and by 28%(*p* < 0.05) at 300 mg/kg. These findings suggest that RO enhances fecal lipid elimination via its fat-binding properties. Blood TG levels were also significantly reduced by RO treatment in a concentration-dependent manner (Fig. 3B). At 150 mg/kg and 300 mg/kg, TG levels decreased by 24% (*p* < 0.05) and 40% (*p* < 0.01), respectively, relative to the lard group (371.8 mg/dL, *p* < 0.001). In DL-treated rats, the 100 mg/kg and 150 mg/kg groups showed no significant difference from the lard group, whereas the 300 mg/kg DL group exhibited a modest but significant 19% increase (*p* < 0.05). However, this was still lower than the 28% observed with RO at the same dose, indicating a relatively weaker fat-binding effect. DL treatment exhibited a weaker effect on fecal fat excretion (Fig. 3C). At 100 mg/kg, 150 mg/kg there was no significant difference compared to the lard group. However, at 300 mg/kg, fecal fat increased by approximately 19% (*p* < 0.05), a modest effect compared to the 28% observed with RO at the same dose. Similarly, DL treatment showed no statistically significant impact on blood TG levels at 100 mg/kg or 150 mg/kg (Fig. 3D). At 300 mg/kg, DL administration resulted in an ~11% reduction in TG, which was not statistically significant.

The RODL (4:1) combination also increased fecal fat excretion in a dose-dependent manner (Fig. 3E). While no significant change was seen at 100 mg/kg, fecal fat increased by 17% (*p* < 0.05) at 150 mg/kg and by 25% (*p* < 0.001) at 300 mg/kg, values comparable to those of RO alone. Blood TG levels were significantly lowered by the RODL combination (Fig. 3F). The combination reduced TG levels by 22% (*p* < 0.01) at 150 mg/kg and by 27% (*p* < 0.001) at 300 mg/kg, relative to the lard group. These results suggest that RO has potent FBC and promotes fecal lipid excretion, leading to improved blood lipid profiles. DL alone showed relatively weaker effects on both blood TG and fecal fat but contributed when combined with RO. The RODL (4:1) combination maintained significant efficacy, indicating a complementary dual mechanism in which RO primarily blocks intestinal fat absorption, while DL contributes to systemic lipid metabolism. This synergy may offer an effective nutritional strategy for managing dietary fat overload and preventing obesity-related complications.

### RO and/or DL Prevent Body Weight Gain in Long-Term HFD-Fed Mice

To evaluate the *in vivo* anti-obesity effects of the combined RO and DL extract, C57BL/6 mice were first fed a HFD for 10 days and then orally administered the RODL mixture (4:1) at various doses daily for 8 weeks, continuing on the HFD. Body weight was recorded every 2–3 days, and animals were sacrificed on day 67 for phenotypic analysis.

**Fig. 4:**
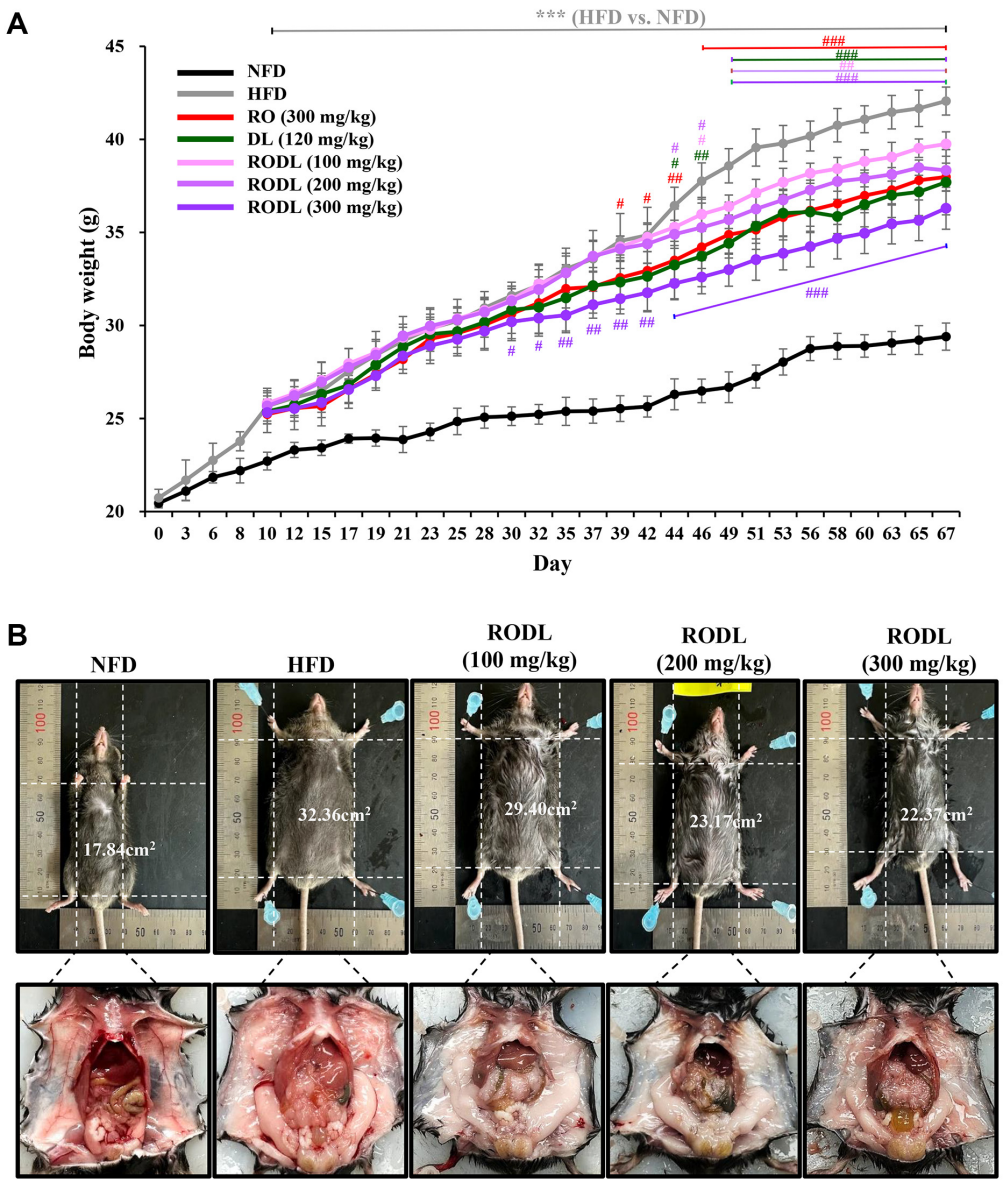
Effects of RO and DL on body weight gain and abdominal adiposity in HFD-fed C57BL/6 mice. After 10 days of high-fat diet (HFD) induction, C57BL/6 mice were orally administered red okra (RO), *D. lotus* (DL), or their combination (RO:DL = 4:1) at varying doses (100, 200, or 300 mg/kg) daily for 8 weeks while continuing HFD feeding. (**A**) Body weight was recorded every 2–3 days throughout the experimental period. (**B**) Representative photographs of sacrificed mice from each group show dorsal view body area measurements (cm^2^) and gross abdominal phenotypes. Data are expressed as mean ± SD (*n* = 16). Statistical significance is denoted as *p < 0.05, **p < 0.01, ***p < 0.001 vs. normal-fat diet (NFD) group; ^#^*p* < 0.05, ^##^*p* < 0.01, ^###^*p* < 0.001 vs. HFD group.

As shown in Fig. 4A, mice fed a HFD exhibited a significant increase in body weight compared to those on a NFD, with this trend persisting throughout the study period (*p* < 0.001). At the end of the experiment, the final body weight of HFD-fed mice reached 42.06 ± 0.75 g, whereas that of NFD-fed mice was 29.40 ± 0.72 g, reflecting a 1.43-fold increase. Oral administration of the RODL combination significantly attenuated HFD-induced weight gain in a dose-dependent manner. Final body weight was reduced by approximately 14% compared to the HFD group. In contrast, treatment with RO (300 mg/kg) or DL (120 mg/kg) alone resulted in approximately 10%reduction in body weight. Notably, the timing of body weight reduction varied across treatment groups. A significant reduction in body weight was observed from day 39 in the RO (300 mg/kg) group, from day 44 in both the DL (120 mg/kg) and RODL (200 mg/kg) groups, and from day 46 in the RODL (100 mg/kg) group. Importantly, in the 300 mg/kg treated RODL group, weight reduction was evident earlier, beginning on day 30. Representative images of mice demonstrated a visibly leaner phenotype in RODL-treated groups compared to HFD controls, with reductions appearing dose-dependent. In contrast, HFD-fed mice exhibited obvious abdominal enlargement and increased overall body mass (Fig. 4B).

Upon dissection, HFD-fed mice displayed marked hypertrophy of subcutaneous and visceral WAT, including eWAT, iWAT, mesenteric WAT (mWAT), anterior subcutaneous WAT (asWAT), and cardiac depots. These adipose tissues were significantly reduced in mice treated with the RODL combination, while interscapular BAT (iBAT) was significantly increased (Table 1). Quantitatively, RO (300 mg/kg) treatment reduced eWAT, iWAT, mWAT, and asWAT weights by 20% (*p* < 0.01), 20% (*p* < 0.05), 33% (*p* < 0.05), and 19% (*p* < 0.05), respectively, and increased iBAT by 41% (*p* < 0.01). DL (120 mg/kg) treatment yielded similar reductions in eWAT (20%, *p* < 0.01), iWAT (23%, *p* < 0.05), mWAT (32%, *p* < 0.05), and asWAT (24%, *p* < 0.05), along with a 27% (*p* < 0.05) increase in iBAT. The RODL combination (300 mg/kg) induced the most pronounced changes, with eWAT, iWAT, mWAT, and asWAT reduced by 21% (*p* < 0.001), 23% (*p* < 0.01), 42% (*p* < 0.01), and 22% (*p* < 0.05), respectively, and iBAT elevated by 60% (*p* < 0.01) relative to HFD controls. Collectively, these findings demonstrate that the RODL combination effectively prevents HFD-induced obesity and adipose tissue expansion. The early onset of body weight reduction and enhanced activation of thermogenic brown fat further support the synergistic anti-obesity potential of this herbal combination.

### Synergistic Effects of RO and DL on Fecal Fat Excretion and Lipid Profiles in HFD-Fed Mice

**Fig. 5:**
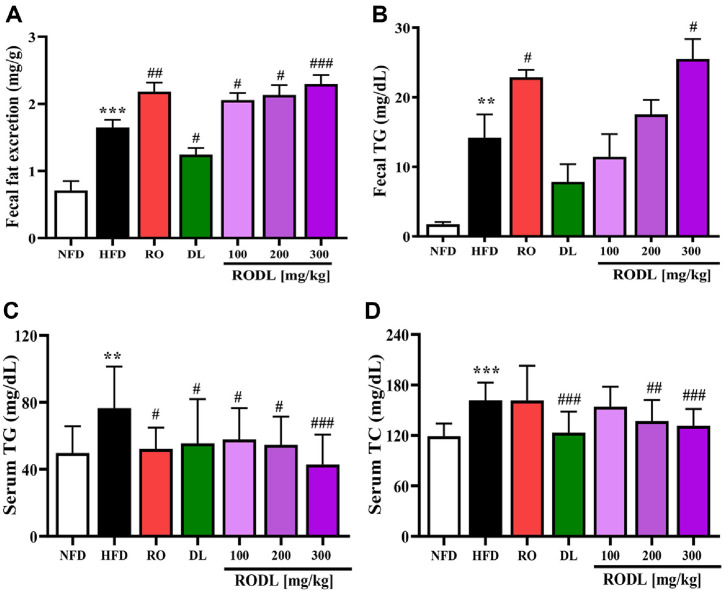
Synergistic effects of RO and DL combination on fecal lipid excretion and serum lipid profiles in HFD-fed mice. C57BL/6 mice were fed a high-fat diet (HFD) for 10 days and subsequently administered red okra (RO) and *D. lotus* (DL) in a fixed 4:1 ratio (RODL) daily for 8 weeks under continued HFD feeding. On the final day of the experiment, fecal and blood samples were collected for lipid analysis. (**A**) Fecal fat content and (**B**) fecal triglyceride (TG) levels were measured to evaluate intestinal lipid excretion. (**C**) Serum TG and (**D**) total cholesterol (TC) levels were assessed to determine systemic lipid profiles. Data are presented as mean ± SD, *n* = 16 per group. Statistical significance is indicated as *p < 0.05, **p < 0.01, ***p < 0.001 vs. normal-fat diet (NFD) group; ^#^*p* < 0.05, ^##^*p* < 0.01, ^###^*p* < 0.001 vs. HFD group.

To evaluate the synergistic effects of RO and DL on lipid metabolism, we analyzed fecal fat content, fecal TG levels, and serum TG and TC in mice subjected to long-term HFD feeding. Fecal fat content (Fig. 5A) was significantly increased in HFD-fed mice compared to NFD controls, showing a 132% (*p* < 0.001) elevation. Treatment with RO (300 mg/kg), DL (120 mg/kg), and RODL combinations at 100, 200, and 300 mg/kg led to further increases in fecal fat by 32% (*p* < 0.01), -25% (*p* < 0.05), 25% (*p* <0.05), 29% (*p* < 0.05), and 39% (*p* < 0.001), respectively, compared to the HFD control group. Notably, while DL alone exerted a modest but significant effect, the RODL (300 mg/kg) group exhibited the most substantial enhancement of fecal fat excretion. Fecal TG content (Fig. 5B) was also dramatically elevated in the HFD control group, by approximately 714% (*p* < 0.01) compared to the NFD group. RO (300 mg/kg) and RODL (300 mg/kg) further increased fecal TG content by 61% (*p* < 0.05) and 80% (*p* < 0.05), respectively, relative to HFD group. However, DL alone (120 mg/kg) and RODL at 100 and 200 mg/kg did not induce significant changes (*p* > 0.05). The fact that RODL (300 mg/kg) caused a greater increase in fecal TG than RO alone suggests a synergistic enhancement of intestinal lipid elimination.

Serum TG levels (Fig. 5C) were elevated by approximately 54% (*p* < 0.01) in the HFD group compared to the NFD group. RO (300 mg/kg), DL (120 mg/kg), and RODL combinations at 100, 200, and 300 mg/kg significantly reduced serum TG by 32% (*p* < 0.05), 27% (*p* < 0.05), 24% (*p* < 0.05), 29% (*p* < 0.05), and 44% (*p* < 0.001), respectively, compared to HFD group. The largest reduction was observed in the RODL (300 mg/kg) group, supporting the presence of a synergistic effect in reducing circulating TG. Serum TC levels (Fig. 5D) were similarly elevated in HFD-fed mice, with a 36% (*p* < 0.001) increase relative to NFD-fed mice. Administration of DL (120 mg/kg), RODL (200 mg/kg), and RODL (300 mg/kg) significantly reduced TC levels by 24% (*p* < 0.001), 15% (*p* < 0.01), and 19% (*p* < 0.001), respectively, compared to HFD group. In contrast, no significant reductions (*p* > 0.05) were observed in the RO (300 mg/kg) and RODL (100 mg/kg) groups.

Taken together, these findings indicate that RO alone exerts strong lipid-lowering and fat-excreting effects, and its combination with DL, particularly at a 4:1 ratio and 300 mg/kg dose, enhances both systemic lipid regulation and intestinal fat elimination. These results support the potential of RO and DL as a synergistic botanical strategy for combating HFD-induced dyslipidemia and promoting lipid homeostasis.

### RO and DL Alleviate HFD-Induced Hepatic Steatosis and Hypertrophy

**Fig. 6:**
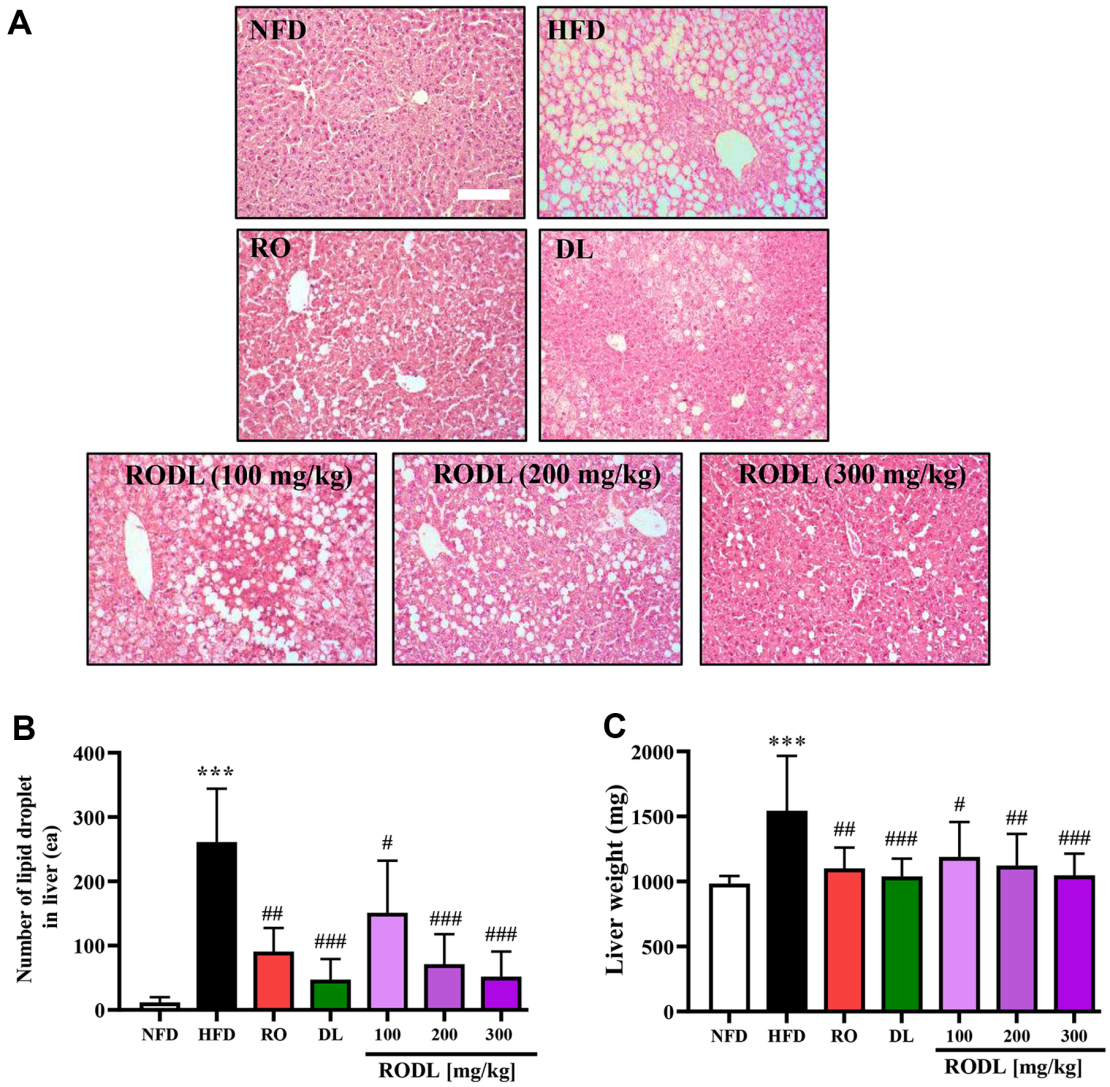
Effects of RO and DL on hepatic steatosis and liver hypertrophy in HFD-fed mice. To evaluate hepatic lipid accumulation, liver tissues from each treatment group were subjected to hematoxylin and eosin (H&E) staining. Mice were fed a normal-fat diet (NFD), high-fat diet (HFD), or treated with red okra (RO), *D. lotus* (DL), or their combination (RODL) at indicated doses (100, 200, or 300 mg/kg) for 8 weeks. (**A**) Representative histological images were captured at 200× magnification; scale bars represent 100 μm. (**B**) The number of lipid droplets per field was quantified to assess steatosis severity. (**C**) Liver weights were measured as an indicator of hepatomegaly. Values are expressed as mean ± SD, n =16 per group. Statistical significance is indicated as *p < 0.05, **p < 0.01, ***p < 0.001 vs. normal-fat diet (NFD) group; ^#^*p* < 0.05, ^##^*p* < 0.01, ^###^*p* < 0.001 vs. HFD group.

Histological analysis of liver sections stained with H&E revealed severe lipid droplet accumulation in the hepatocytes of HFD-fed mice, indicating advanced hepatic steatosis (Fig. 6A). In contrast, treatment with RO, DL, or their combination at a 4:1 ratio (RODL) markedly attenuated lipid accumulation. Among the single extract treatments, RO showed greater efficacy in reducing hepatic lipid deposition compared to DL. Notably, the RODL combination further improved hepatic histology in a dose-dependent manner, with the 300 mg/kg group exhibiting the most pronounced reduction in hepatic fat vacuolation and near-normal liver architecture (Fig. 6A and 6B). Quantitative assessment of liver weight, an indicator of steatosis-related hepatomegaly, supported the histological findings (Fig. 6C). Liver weight in the HFD group was significantly elevated compared to the normal-fat diet (NFD) group (*p* < 0.001). Treatment with RO, DL, and RODL significantly reduced liver weight relative to the HFD group. Among these, the RODL combination showed the most substantial reduction at the 300 mg/kg, suggesting a dose-related improvement in hepatic hypertrophy.

In addition to histological improvements, extract administration also modulated key biochemical markers (Table 2). LDL, a key indicator of hypercholesterolemia, was significantly elevated in the HFD group. DL and RODL (300 mg/kg) treatments significantly reduced LDL levels, while RO alone had no significant effect. Markers of hepatic damage and metabolic dysfunction, including ALT, LDH, and glucose, were significantly increased in the HFD group. These elevations were significantly attenuated by the RODL (300 mg/kg) treatment. In contrast, RO or DL alone showed only partial, non-significant reductions. These findings further support the hypothesis that RO and DL modulate lipid metabolism through distinct mechanisms. While RO appears to exert stronger effects on hepatic lipid accumulation and liver weight, DL shows superior efficacy in lowering circulating LDL levels. Their combination achieves complementary benefits, suggesting that a 4:1 RODL mixture may serve as an effective strategy to simultaneously target multiple pathways involved in HFD-induced hepatic and metabolic disorders.

### RODL Combination Enhances Lipid Metabolism via Lipolysis, Thermogenesis, and FA Oxidation in Epididymal White Adipose Tissue (eWAT)

**Fig. 7:**
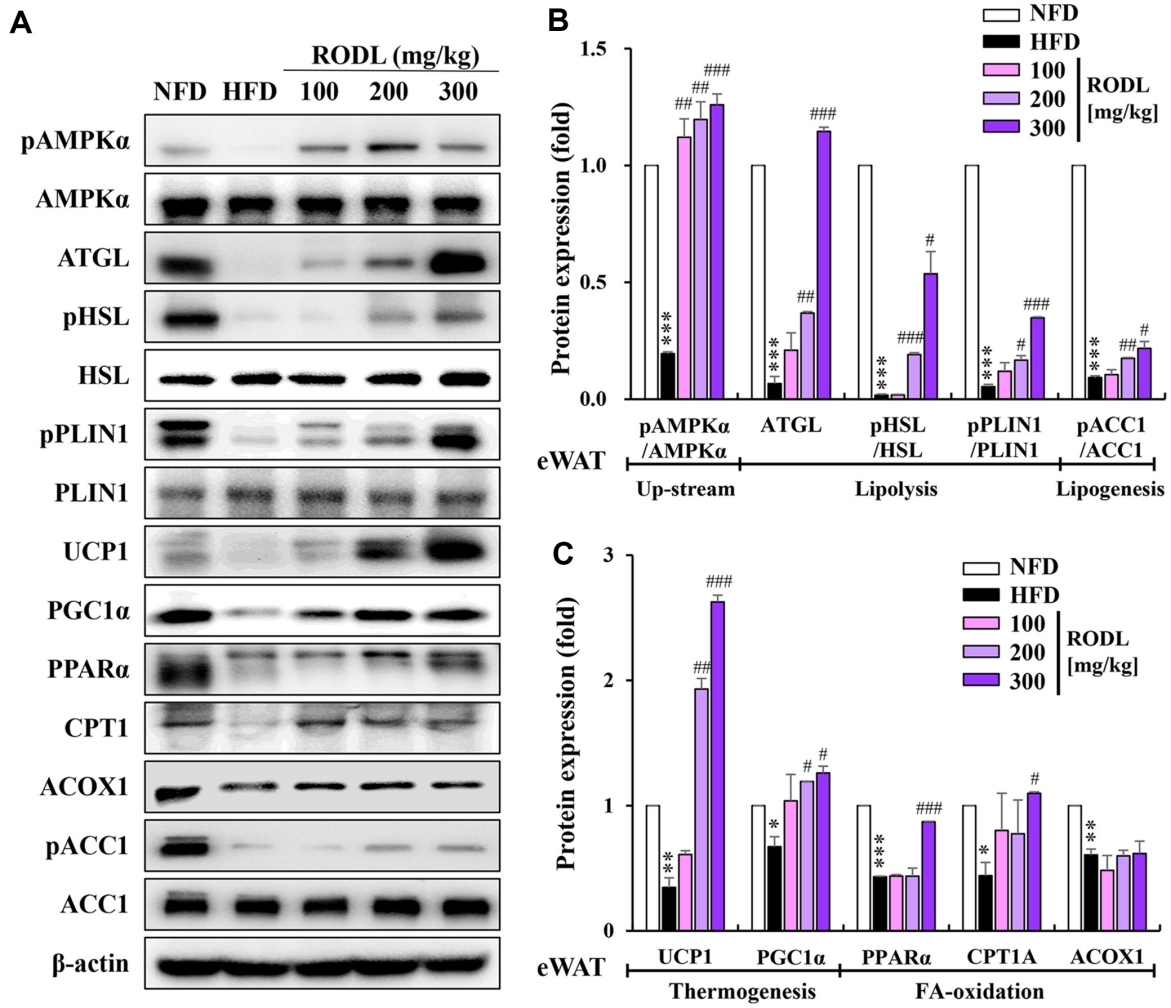
Effects of RODL combination on lipid metabolism-associated proteins in epididymal white adipose tissue (eWAT) of HFD-fed mice. Western blot analysis was performed to examine the expression of key regulators of lipid metabolism in eWAT from C57BL/6 mice administered a red okra (RO) and *D. lotus* (DL) mixture (RODL) at 100, 200, or 300 mg/kg under high-fat diet (HFD) conditions. Protein levels of phosphorylated AMP-activated protein kinase α (pAMPKα), total AMPKα, adipose triglyceride lipase (ATGL), phosphorylated and total hormone-sensitive lipase (pHSL and HSL), phosphorylated and total perilipin 1 (pPLIN1 and PLIN1), uncoupling protein 1 (UCP1), peroxisome proliferatoractivated receptor γ coactivator 1α (PGC1α), PPARα, carnitine palmitoyltransferase 1 (CPT1), acyl-coenzyme A oxidase 1 (ACOX1), phosphorylated acetyl-CoA carboxylase 1 (pACC1), and total ACC1 were evaluated (**A**). Protein expression levels were normalized to β-actin, and phosphoprotein signals were normalized to their respective total protein levels. Quantitative densitometric analyses are shown in (**B**) and (**C**). Data are presented as mean ± SD from three independent experiments. *p < 0.05, **p < 0.01, ***p < 0.001 vs. normal-fat diet (NFD); ^#^*p* < 0.05, ^##^*p* < 0.01, ^###^*p* < 0.001 vs. HFD.

Western blot analysis of eWAT revealed that the expression of key lipolytic regulators, including ATGL, pHSL, and pPLIN1, was markedly reduced in HFD-fed mice. However, this suppression was significantly reversed in a dose-dependent manner following administration of the RODL (4:1) combination. Additionally, the pACC1, indicative of reduced lipogenesis, was significantly increased upon RODL treatment. Notably, the RODL combination also restored thermogenic markers such as UCP1 and PGC1α, which were suppressed in the HFD group. These effects were most pronounced at doses of 200 and 300 mg/kg. Furthermore, RODL (300 mg/kg) treatment significantly increased the expression of PPARα and CPT1, which are key regulators of mitochondrial β-oxidation. In contrast, ACOX1, a peroxisomal FA oxidation enzyme, was not significantly altered by treatment. Importantly, pAMPKα, a master regulator of lipid metabolism, was markedly reduced in the HFD group but significantly restored by RODL administration at all tested doses, suggesting reactivation of AMPK signaling in eWAT (Fig. 7A-7C).

### RODL Combination Promotes Thermogenesis and Fatty Acid Oxidation in Inguinal White Adipose Tissue (iWAT)

**Fig. 8:**
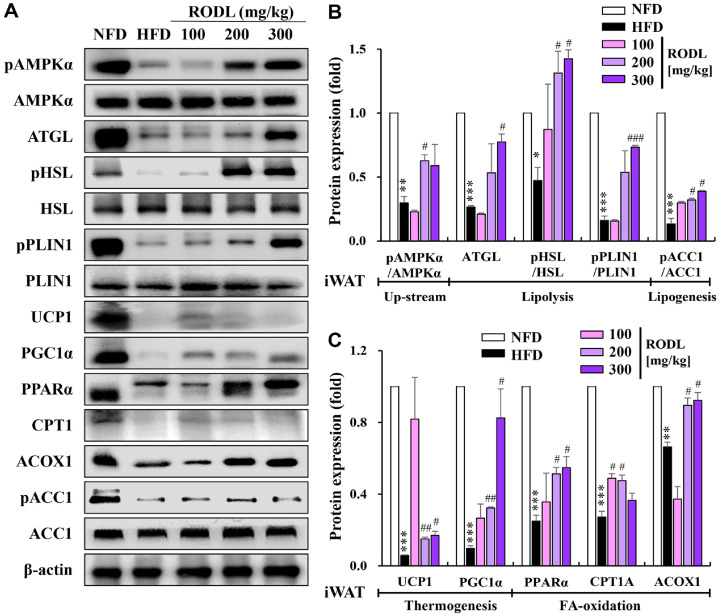
Effects of RODL combination on lipid metabolism-associated proteins in inguinal white adipose tissue (iWAT) of HFD-fed mice. Western blot analysis was conducted to assess the expression of key proteins involved in lipid metabolism in the inguinal white adipose tissue (iWAT) of mice fed a high-fat diet (HFD) and treated with a red okra (RO) and *D. lotus* (DL) combination (RODL) at 100, 200 or 300 mg/kg for 8 weeks. The analyzed proteins included phosphorylated and total forms of AMP-activated protein kinase α (pAMPKα and AMPKα), adipose triglyceride lipase (ATGL), hormonesensitive lipase (pHSL and HSL), perilipin 1 (pPLIN1 and PLIN1), uncoupling protein 1 (UCP1), peroxisome proliferatoractivated receptor γ coactivator 1α (PGC1α), PPARα, carnitine palmitoyltransferase 1 (CPT1), acyl-coenzyme A oxidase 1 (ACOX1), phosphorylated acetyl-CoA carboxylase (pACC1), and total ACC1 (**A**). Densitometric analysis was performed to quantify relative protein expression (**B, C**), normalized to β-actin for total proteins and to corresponding total protein levels for phosphoproteins. Data are presented as mean ± SD from three independent experiments. *p < 0.05, **p < 0.01, ***p < 0.001 vs. normal-fat diet (NFD); ^#^*p* < 0.05, ^##^*p* < 0.01, ^###^*p* < 0.001 vs. HFD group.

In iWAT, HFD feeding significantly suppressed the expression of thermogenesis-related proteins, including UCP1 and PGC1α. RODL treatment at 200 mg/kg and higher significantly reversed this suppression. A similar dose-dependent restoration was observed in lipolytic markers ATGL, pHSL, and pPLIN1. Additionally, the expression of PPARα and ACOX1, which were significantly reduced in HFD-fed mice, was significantly increased in the RODL treatment groups at 200 and 300 mg/kg. CPT1 expression was also significantly elevated at 100 and 200 mg/kg, indicating enhanced mitochondrial fatty acid oxidation. Interestingly, pAMPKα levels were significantly increased only at the 200 mg/kg dose, suggesting a dose-dependent activation of AMPK signaling in iWAT (Fig. 8A-8C).

### RODL Combination Induces Thermogenic Activation in Brown Adipose Tissue (BAT)

**Fig. 9:**
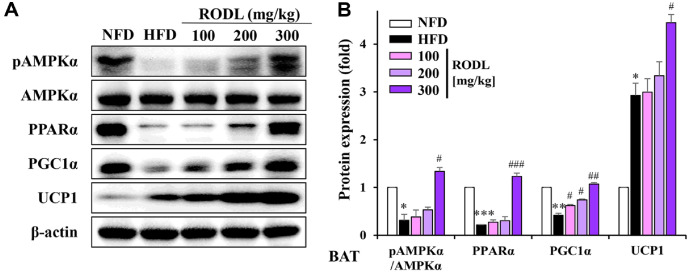
Effects of RODL combination on lipid metabolism-related proteins in brown adipose tissue (BAT) of HFD-fed mice. Western blot analysis was performed to evaluate the expression of lipid metabolism-related proteins in BAT of C57BL/6 mice fed a high-fat diet (HFD) and treated with red okra (RO) and *D. lotus* (DL) (RODL) at 100, 200, or 300 mg/kg for 8 weeks. Protein levels of phosphorylated and total AMP-activated protein kinase α (pAMPKα, AMPKα), peroxisome proliferator-activated receptor α (PPARα), PGC1α, and uncoupling protein 1 (UCP1) were assessed (A). Densitometric quantification is shown in (B), where expression was normalized to β-actin and phosphoproteins were normalized to their respective total protein levels. Data are presented as mean ± SD from three independent experiments. Statistical significance is denoted as *p < 0.05, **p < 0.01, ***p < 0.001 vs. normal-fat diet (NFD); ^#^*p* < 0.05, ^##^*p* < 0.01, ^###^*p* < 0.001 vs. HFD group.

The thermogenic activity of BAT was significantly enhanced by RODL treatment, as evidenced by the upregulation of UCP1 protein expression in Western blot analyses. Compared to the HFD group, RODL administration markedly increased UCP1 levels, indicating activation of thermogenesis. Additionally, the expression of mitochondrial biogenesis-associated transcription factors, PGC1α and PPARα, was significantly elevated. These effects were accompanied by a concurrent increase in pAMPKα levels, suggesting that the observed thermogenic activation in BAT is mediated, at least in part, by AMPK signaling. Together, these findings demonstrate that the RODL combination not only stimulates BAT activity but also contributes to the browning of white adipose tissue depots (Fig. 9A and 9B).

### Standardization of RO and DL Extracts via Repetitive Quantitative HPLC Analysis

To ensure batch-to-batch consistency and chemical standardization of the RO and DL used in this study, repeated extractions and HPLC-based quantification of their respective marker compounds, quercetin-3-O-gentiobioside for RO and myricetin for DL, were performed. Each extract was independently prepared five times under identical extraction conditions, and the concentrations of the marker compounds were quantified using a validated HPLC method.

**Fig. 10:**
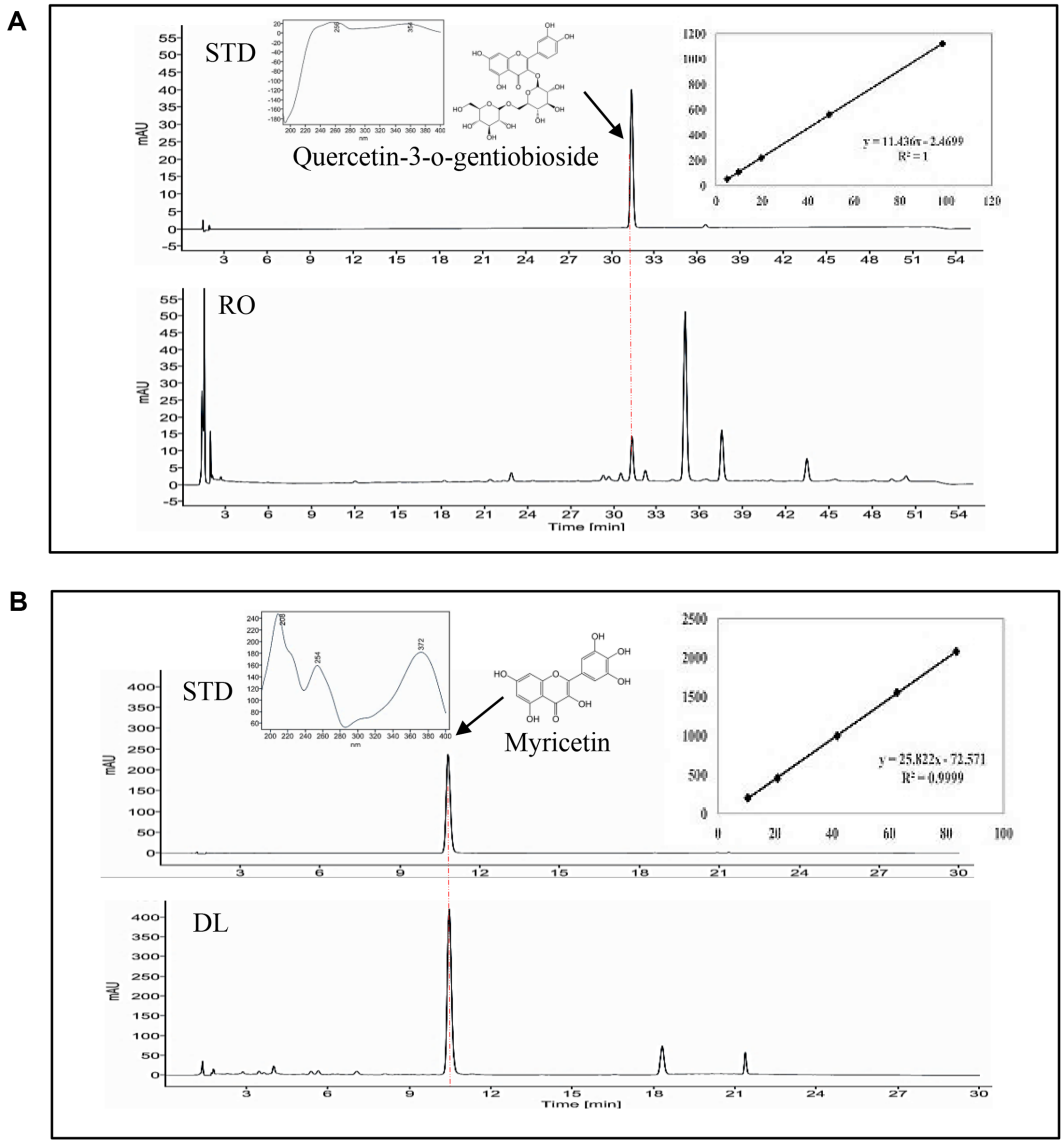
Quantitative HPLC analysis of quercetin-3-O-gentiobioside and myricetin in RO and DL. High-performance liquid chromatography (HPLC) was used to determine the contents of quercetin-3-O-gentiobioside in red okra (RO) and myricetin in *D. lotus* (DL). (**A**) Representative chromatogram of standard quercetin-3-O-gentiobioside with retention time 31.36 min, along with UV-Vis spectral profile and calibration curve. Corresponding peak identified in RO, with quantification expressed as mg/g of dry extract. (**B**) HPLC chromatogram of standard myricetin with retention time 10.45 min, showing UV absorption and standard curve. Myricetin peak in DL was identified and quantified using the same conditions. The chromatographic analysis was performed using a reversed-phase C18 column, with detection wavelengths set at 354 nm for quercetin-3-O-gentiobioside and 370 nm for myricetin. Retention time and UV spectra were matched with those of authentic standards to confirm compound identity. Data are presented as mean ± SD.

As shown in Fig. 10, quercetin-3-O-gentiobioside in RO was consistently detected across five independent preparations, yielding concentrations of 2.07 ± 0.15 mg/g. Similarly, the myricetin content in DL was quantified at 20.48 ± 1.90 mg/g. The relative standard deviations (RSDs) for both compounds were within acceptable analytical limits (<10%), indicating high reproducibility and robustness of both the extraction and analytical procedures.

These findings confirm that the RO and DL are chemically standardized with respect to their representative bioactive constituents. The consistent quantification of quercetin-3-O-gentiobioside and myricetin across multiple batches substantiates the suitability of these extracts as reliable and reproducible materials for subsequent pharmacological evaluations.

## Discussion

Obesity is a major public health concern that contributes to the development of various chronic diseases, including metabolic syndrome, cardiovascular disease, and type 2 diabetes. Although lifestyle interventions such as caloric restriction and physical activity remain foundational strategies for weight management, adherence is often limited in real-world settings. Accordingly, natural products have gained increasing attention as complementary or alternative therapies for obesity, particularly for individuals who are unable to comply with conventional regimens.

In the present study, we demonstrated that a combined extract of RO and DL, administered at a fixed 4:1 ratio, effectively ameliorated HFD-induced obesity in mice via a dual mechanism: (i) inhibition of dietary fat absorption through enhanced fat-binding capacity, resulting in increased fecal lipid excretion; and (ii) activation of AMPKα-mediated intracellular lipid metabolism, which promoted lipolysis, thermogenesis, and FA oxidation. This dual action strategy provides both peripheral and systemic anti-obesity effects, highlighting the synergistic potential of RO and DL when co-administered.

Previous studies have shown that green okra exerts anti-obesity effects by suppressing adipogenesis and enhancing thermogenic activity through PKA/UCP1 signaling in HFD-fed mice [19, 20]. Our findings extend this understanding by showing that red okra, in addition to modulating thermogenic signaling, possesses potent fat-binding capacity that contributes to lipid sequestration in the gastrointestinal tract. Notably, RO alone significantly increased fecal lipid excretion and activated AMPKα signaling and lipolytic proteins such as ATGL and pHSL. These effects support its dual role in both physical fat capture and metabolic activation.

Clinical evidence also corroborates the gut-level mechanism of okra. A randomized, double-blind clinical trial using IQP-AE-103 (a green okra and inulin complex) reported significant weight reduction and favorable shifts in gut microbiota, including increased *Akkermansia muciniphila*, implicating a microbiota-mediated component in fat absorption and energy balance [21, 22].

In parallel, DL extract has previously been reported to attenuate weight gain, hyperlipidemia, and hepatic steatosis in HFD-fed mice while enhancing antioxidant defenses [23]. Consistent with these findings, our data demonstrate that DL alone enhances lipolysis, as evidenced by increased glycerol release and upregulation of ATGL and pHSL in adipocytes. When combined with RO, DL synergistically enhanced AMPKα phosphorylation and further stimulated downstream lipolytic and oxidative signaling pathways.

Our study also aligns with existing literature on other botanical extracts such as *Nelumbo nucifera* (lotus leaf), *Rosa acicularis*, and *Diospyros kaki*, which have been shown to activate AMPK signaling, suppress adipogenesis, and enhance thermogenesis and FA oxidation [21, 24, 25]. Apigenin, a dietary flavonoid, similarly upregulates UCP1 and PGC1α through AMPK-ACC signaling, reinforcing the concept that many natural compounds act through shared metabolic pathways [21]. However, the unique aspect of our study lies in the mechanistic complementarity of RO and DL, RO acts predominantly at the gastrointestinal level, while DL exerts intracellular regulatory effects, together establishing a holistic anti-obesity intervention.

Compared to chitosan, a widely studied fat-binding agent, RO exhibits comparable or superior fat-binding capacity (~40 g of fat/g powder), with the added advantage of AMPKα-mediated metabolic activation. Clinical trials of chitosan have reported modest effects on body weight and serum lipids, often hindered by poor adherence and gastrointestinal side effects [26, 27]. Although chitosan can modulate gut microbiota and intestinal barrier function, its intracellular signaling impact remains limited [28]. In contrast, the RODL combination simultaneously promotes lipid sequestration and metabolic reprogramming, resulting in robust reductions in body weight (~14%), serum triglycerides (~44%), and hepatic steatosis. These findings support its superiority over single-mechanism agents such as chitosan.

Mechanistically, the extract combination significantly enhanced lipolytic gene expression in white adipose tissues (eWAT and iWAT), including ATGL, pHSL, and pPLIN1, facilitating the mobilization of stored triglycerides. Glycerol release assays further confirmed functional activation of lipolysis, consistent with AMPK-dependent regulation [29, 30]. In addition, upregulation of UCP1 and PGC1α in both white and brown adipose tissues suggests that the combination extract promotes browning of WAT and enhances thermogenic capacity, a mechanism associated with improved insulin sensitivity and lipid clearance [31, 32].

Following lipolysis, efficient oxidation of FFAs is crucial for energy production and prevention of lipotoxicity. The extract significantly induced mitochondrial and peroxisomal FA oxidation via increased expression of PPARα, CPT1, and ACOX1. CPT1 serves as a rate-limiting enzyme for mitochondrial fatty acid transport, while ACOX1 initiates peroxisomal β-oxidation of very-long-chain fatty acids [33-35]. Upregulation of these genes suggests comprehensive activation of oxidative pathways, providing metabolic flexibility and reducing ectopic fat accumulation. Restoration of CPT1 and PPARα activity is particularly relevant in obesity and diabetes, where mitochondrial oxidation is often impaired [36, 37].

Collectively, the coordinated activation of AMPKα, lipolysis, thermogenesis, and FA oxidation indicates a shift from lipid storage to lipid utilization, a profile reminiscent of exercise-induced metabolic remodeling. These integrated effects position the RODL mixture as a potent metabolic activator, surpassing the efficacy of single-agent botanical or pharmacological interventions. The consistent upregulation of these pathways in both WAT and BAT further supports the systemic metabolic reprogramming induced by the extract [30, 38].

Despite these promising findings, translational studies in humans remain necessary. Pharmacokinetic analyses, long-term safety profiling, and validation through transcriptomic or proteomic approaches will be critical to establish therapeutic relevance. Knockdown studies targeting AMPK or its downstream effectors may further elucidate the causal pathways. Ultimately, clinical trials are warranted to confirm the safety, efficacy, and dosing of the RODL combination in obese human populations.

## Conclusion

This study demonstrates that a combined extract of RO and DL exerts potent anti-obesity effects through a synergistic dual mechanism. Specifically, the mixture simultaneously inhibits dietary fat absorption via enhanced fat-binding capacity and activates AMPK-dependent lipid metabolic pathways, thereby promoting lipolysis, thermogenesis, and fatty acid oxidation. This integrated mechanism underscores the therapeutic potential of the RODL combination as a safe and effective botanical intervention for the prevention and management of obesity and associated metabolic disorders. The extract combination ratio (RO:DL = 4:1) and dose selection (100–300 mg/kg) were determined based on preliminary *in vitro* screening of fat-binding activity and lipid accumulation assays. While these doses showed efficacy *in vivo*, future studies should further optimize the dosing range and investigate the pharmacokinetic behavior of the active compounds.

Additionally, while no signs of acute toxicity were observed in the current models, chronic toxicity and long-term safety assessments, including liver, kidney, and hematological parameters, will be essential for translational development. Future research should also examine potential interactions with gut microbiota and explore clinical relevance through human trials.

## Supplemental Materials

Supplementary data for this paper are available on-line only at http://jmb.or.kr.



## Figures and Tables

**Table 1:** Effect of RO and DL on Tissue Weights in HFD-Fed Mice.

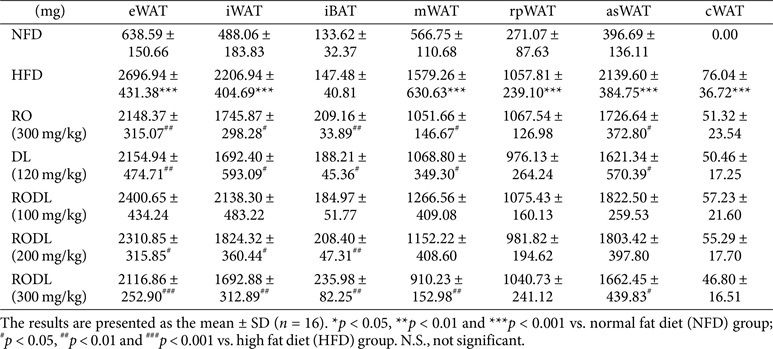

**Table 2:** Effect of RP and DL on Blood Biochemical Parameter in HFD-Fed Mice.