# Supplementary material for: Dual Mechanistic Anti-Obesity Effects of Red Okra and Diospyros lotus Mixture via Fat Binding and AMPK-Mediated Lipid Metabolism
Source: J Microbiol Biotechnol. 2025 Sep 24;35:e2506030. doi: 10.4014/jmb.2506.06030 (PMC12535863; doi:10.4014/jmb.2506.06030)
Supplement: Supplementary file 1 [file jmb-35-e2506030-supple.pdf]

J. Microbiol. Biotechnol. 2025. 35: e2506030

https://doi.org/10.4014/jmb.2506.06030

Method

Cell viability assay (MTT assay)

The cytotoxicity of red okra (RO) and *D. lotus* (DL) was evaluated in 3T3-L1 preadipocytes using the MTT assay. Cells were seeded in 96-well plates at a density of 1 × 10^4 cells/well and incubated for 24 h at 37 °C in a humidified atmosphere containing 5% CO_2. Cells were then treated with increasing concentrations (0, 5, 10, 50, 100, and 200 μg/ml) of RO or DL for 24 h. After treatment, 10 μL of MTT solution (5 mg/ml) was added to each well and incubated for 4 h. The resulting formazan crystals were dissolved in 100 μl of DMSO, and absorbance was measured at 570 nm using a microplate reader. Cell viability was expressed as a percentage relative to untreated control cells. All experiments were conducted in triplicate.

Supplementary Figure

Fig. S1. Effects of red okra and diospyros extracts on 3T3-L1 cell viability.

3T3-L1 preadipocytes were treated with varying concentrations (0–200 μg/ml) of red okra (RO) or *D. lotus*(DL) for 24 h. Cell viability was determined using the MTT assay. Data are presented as mean ± standard deviation (SD) from three independent experiments. No significant cytotoxicity was observed up to 200 μg/ml for any treatment group, confirming the safety of the test extracts for subsequent *in vitro* assays. N.S., not significant vs. control (con).

A

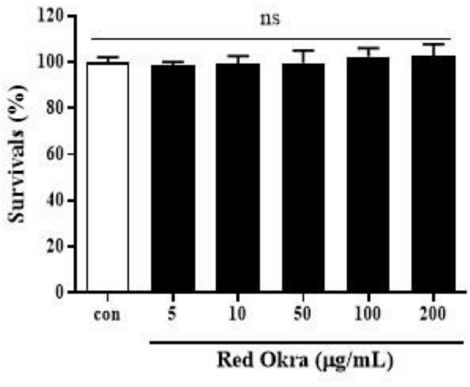

ns
Survivals (%)
120
100
80
60
40
20
0
con
5
10
50
100
200
Red Okra (μg/mL)

B

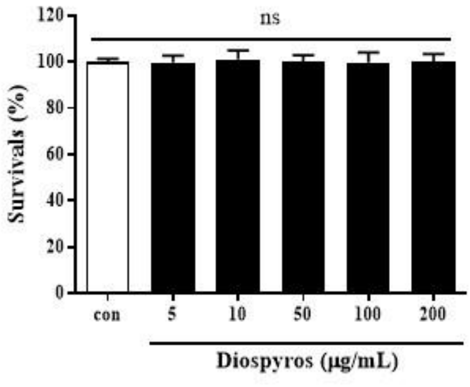

ns
Survivals (%)
120
100
80
60
40
20
0
con
5
10
50
100
200
Diospyros (μg/mL)
